# Single-cell atlas of the developing mouse oviduct reveals smooth muscle heterogeneity and identifies Vps34 as a critical regulator of female fertility

**DOI:** 10.1186/s12915-026-02706-4

**Published:** 2026-08-07

**Authors:** Yanhua He, Yong Zhang, Yunxia Chen, Lefan Ju, Wen Fang, Qian Huang, Xiaoyan Ying, Wenwen Liu, Xiaowei Dou

**Affiliations:** 1 Department of Obstetrics and Gynecology, The Second Affiliated Hospital of Nanjing Medical University, Nanjing, China; 2 Department of Obstetrics and Gynecology, Affiliated Hospital of Medical School, Jinling Hospital, Nanjing University, Nanjing, China; 3 The Second Clinical Medical College, Nanjing Medical University, Nanjing, China; 4 Department of Prenatal Diagnosis, Women’s Hospital of Nanjing Medical University, Nanjing Women and Children’s Healthcare Hospital, Nanjing, China

**Keywords:** Oviduct, Smooth muscle cells, Single-cell RNA sequencing, Vps34, Female fertility

## Abstract

**Background:**

The oviduct smooth muscle layer is traditionally regarded as a contractile unit. Limited understanding exists regarding the molecular heterogeneity and intercellular communication during development timeline, which is critical for female reproductive function. To address this, we performed single-cell RNA sequencing (scRNA-seq) to profile the cellular composition of mice oviducts at four developmental stages: neonatal (7 days), pre-pubertal (3 weeks), young adult (8 weeks) and middle-aged (9 months). We aim to provide a framework for further investigation of oviduct biology and associated reproductive disorders.

**Results:**

We delineate three distinct (SMC) subtypes, with their relative proportions shifting dynamically across the developmental timeline. SMC1 represented a transient progenitor population exclusive to neonatal oviducts. SMC2 displayed a mature contractile phenotype, while SMC3 uniquely expressed hormone-responsive genes (Esr1, Pgr) and showed enrichment of autophagy-related pathways. Functional studies demonstrated that SMC-specific deletion of Vps34 disrupts oviduct coiling and muscular integrity, leading to vacuolar degeneration and a significant reduction in litter size in mice. Additionally, we map five ciliated and six secretory epithelial subtypes and suggest that FN1 signaling may serve as a potential mediator of crosstalk between epithelial and SMCs during development, with the FN1-SDC4 axis emerging as the dominant interaction in mature oviducts. Thus, while the contractile and hormone-responsive functions of the oviductal muscle layer have long been recognized, our work provides its underlying cellular complexity during development.

**Conclusion:**

Our study constructs a developmental atlas of murine oviduct SMCs, revealing that the myosalpinx is composed of functionally distinct SMC subtypes with dynamic developmental trajectories. The identification of Vps34 as a critical guardian of myosalpinx integrity offers a new cellular framework for understanding human tubal pathologies. These findings highlight the importance of SMC subtype homeostasis and autophagy-mediated maintenance of myosalpinx structure, providing a basis for future mechanistic studies on oviduct function and dysfunction.

**Supplementary Information:**

The online version contains supplementary material available at https://doi.org/10.1186/s12915-026-02706-4.

## Background

The oviduct, also called Fallopian tube (FT) in humans, is a highly specialized organ responsible for gamete transport, fertilization, and early embryo development, which plays a vital role in women’s reproductive function [1]. Its ability to efficiently transport the embryo towards the uterus is fundamental for successful pregnancy [2]. Abnormal pregnancies such as ectopic pregnancies (EP) could occur due to disorders in tubal motility caused by inflammation, infection, endometriosis, or maternal cigarette smoking [3]. Histologically, the entire tube is composed of three layers: an innermost epithelial/mucosal layer, bounded internally by a central muscular layer composed predominantly of smooth muscle cells (SMCs), and an outer surface surrounded by serosa [4]. The dynamic transport function of the oviduct is achieved through coordinated ciliary beating of the epithelium and peristaltic contractions of the smooth muscle layer, picking up the oocyte after ovulation and propelling the embryo toward the uterine cavity [5, 6].

It was reported that oviductal motile cilia are essential for oocyte pickup but dispensable for sperm and embryo transport, suggesting smooth muscle contraction may be the major force driving the embryo transport [7]. Indeed, studies in mouse models have shown that disruption of oviductal cilia function does not completely block fertility [8], and similar observations have been reported in women with Kartagener syndrome [2, 9, 10], whereas in patients with EP, the spontaneous rhythmic contraction of SMCs was markedly attenuated [3]. Despite the critical importance of oviductal smooth muscle layers in fertility and pathologies like EP, their developmental trajectory, molecular identity, and functional maturation remain poorly characterized. Recently, single-cell techniques have quickly expanded our understanding of FT and its associated diseases [1, 4, 11, 12], and also revealed cellular diversity and gene expression in different phases, such as the menstrual cycle or menopausal status [5]. Although these reports provided valuable insights into the epithelial landscape of the FT, the specific cellular makeup of its smooth muscle cells remains unexplored. Moreover, current researches predominantly focus on the FT in adult or perimenopausal and postmenopausal subjects, lacking systematic investigation into dynamic changes across the developmental continuum.

To address this critical knowledge gap, our study defines the developmental timeline and cell characteristics of oviductal SMCs using a murine model. We employed single-cell RNA sequencing (scRNA-seq) to map the cellular composition of the mouse oviduct across four representative developmental stages: neonatal (7 days), pre-pubertal (3 weeks), young adult (8 weeks), and middle-aged (9Months). Our results revealed distinct phases of SMCs maturation. Specifically, precursor SMCs dominate the neonatal oviduct. As development progresses (3 weeks, 8 weeks, 9Months), these precursors transition, giving rise to defined populations of contractile SMCs and hormone-responsive SMCs. Notably, the relative proportions of these smooth muscle cell (SMC) subtypes shifted dynamically across developmental stages.Then, we identified Vps34 as a central protective factor for the development and functions of the oviduct. Our work will advance beyond the 'epithelium-centric' paradigm in oviduct research by establishing the first atlas of smooth muscle cells (SMCs) associated with oviduct motility disorders. We not only dissected SMC heterogeneity and identified Vps34 as a key regulator of female fertility, but also uncovered a dynamic SMC-epithelial crosstalk mediated by FN1 signaling, providing a foundation for understanding how these two layers coordinate during development. This foundational resource will provide a foundational cellular framework for understanding tubal physiology and pathologies such as tubal factor infertility.

## Results

### Cellular composition of oviducts and the relative proportions in different developmental stages

**Fig. 1:**
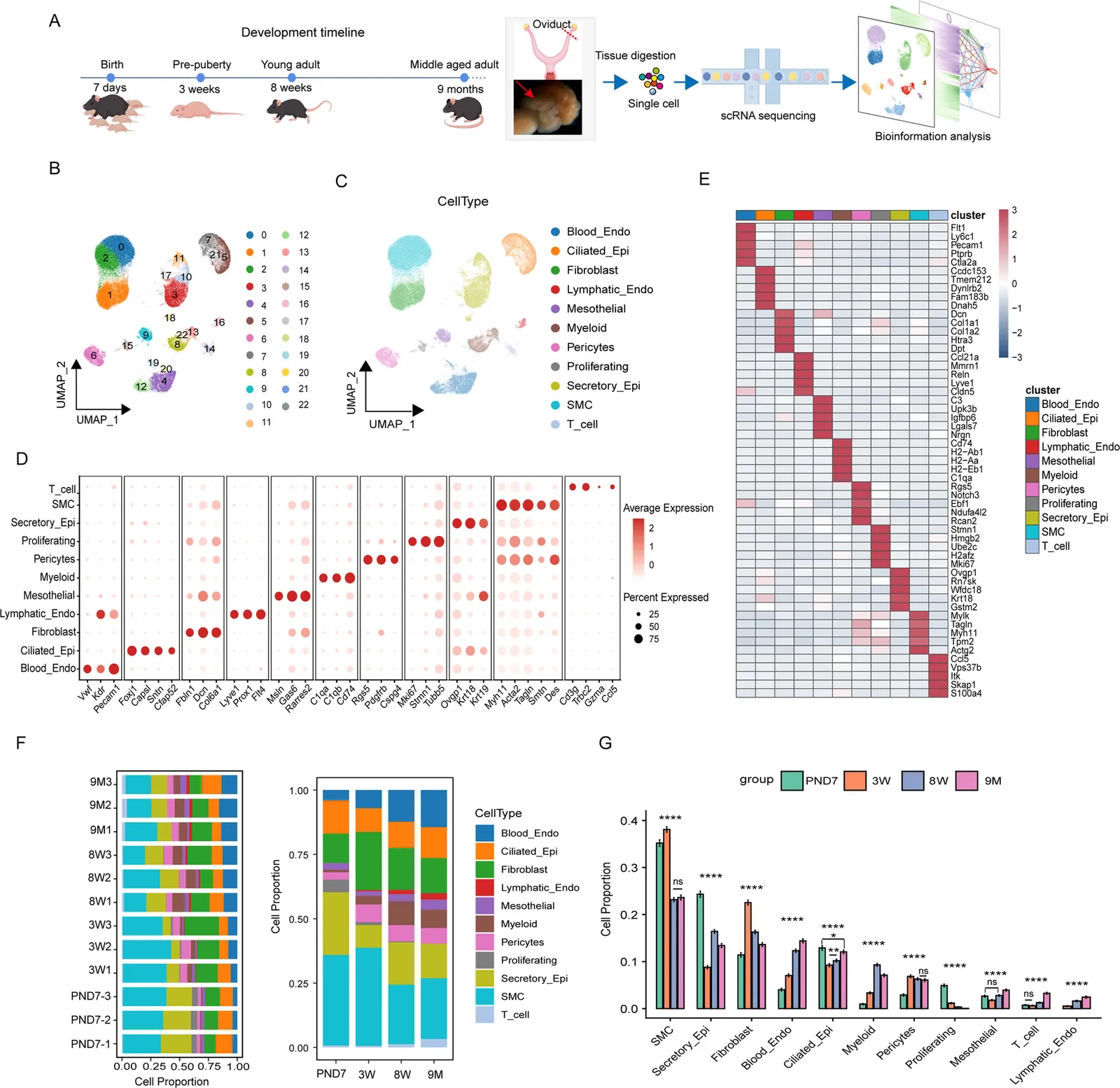
Single-cell transcriptome profiling of mice oviduct. **A** The study flowchart. Mouse oviducts were collected at PND7, 3W, 8W, and 9M for scRNA-seq. Three biological replicates were prepared per time point: for PND7, 6 mice per replicate; for 3W, 8W and 9M, 4 mice per replicate. **B-C** Identification of 11major cell types from global clustering of cells from mouse oviducts, visualized in UMAP space. **D** Dot plot heat map showing normalized gene expression levels of marker genes in each cell population. **E** Heatmap of top 5 differentially expressed genes in the 11 major cell subpopulations. **F** Cell proportion distributions of 11 clusters in each samples and stages from PND7, 3W, 8W, and 9M. **G** Quantification of cell type proportions at PND7, 3W, 8W, and 9M. Data are shown as mean ± SD (*n* = 3 biological replicates per time point). The Chi-square test was used for pairwise comparisons: PND7 vs. 3W, 3W vs. 8W, PND7 vs. 8W, 9M vs. 8W, 9M vs. PND7, and 9M vs. 3W. Significance levels after Bonferroni correction: ****P < 0.0001 indicates that all six pairwise comparisons for a given cell type, **P < 0.01, *P < 0.05, “ns” indicates no significant difference in a specific comparison

To capture critical developmental transitions, oviducts was sampled at postnatal day 7 (PND7), coinciding with the completion of initial differentiation [13]. Subsequent timepoints at 3 weeks (pre-puberty, 3W), 8 weeks (sexually mature, 8W), and 9Months (onset of reproductive senescence, 9M) were selected to encompass major functional stages and aging. The overview of the study design for the oviduct developmental atlas is shown in Fig. 1A. In total, we profiled 109,923 single cells isolated from the oviducts of all samples. Following standard quality control metrics to remove low-quality cells, doublets, and potential erythrocyte contamination, we generated a high-quality dataset comprising 102,420 single-cell transcriptomes for all subsequent analyses (Additional file 1: Fig. S1A-C; Additional file 2: Tab. S1). We identified 23 cell clusters using the Uniform Manifold Approximation and Projection (UMAP) algorithm for the nonlinear dimensionality reduction analysis (Fig. 1B), and categorized them into 11 major cell types based on the expression of canonical marker genes (Fig. 1C-D and Additional file 1: Fig. S1D; Additional file 3: Tab. S2). All major clusters could be detected in each of the samples analyzed (Additional file 1: Fig. S1C), of which, smooth muscle cells (SMCs) accounted for the majority of cells, and feature high expression of Myh11, Acta2, Tagln and Des [5] (Fig. 1D, F, G and Additional file 1: Fig. S1D). Epithelium cells has two distinct clusters: ciliated_Epi (CEs) and secretory _Epi (SEs) with specifical expression of Foxj1 and Ovgp1 (Fig. 1D and Additional file 1: Fig. S1D), respectively, which has been well reported in the previous study [11]. Notably, Fig. 1E further displays the top 5 differentially expressed genes (DEGs; |avg_logFC|> 0.25 and Padj < 0.05) for each cell subcluster, which distinctly characterize the molecular identity of each cell type. Concurrently, we detected a marked expansion of immune populations, specifically T cells and myeloid cells. Furthermore, the proportions of vascular endothelial cells (Blood_Endo) and lymphatic endothelial cells (Lymphatic_Endo) demonstrated a consistent and gradual increase along the developmental timeline (Fig. 1F-G). In contrast, proliferating cells, although a minor population at all time points, exhibited a pronounced decrease in their relative fraction, becoming nearly undetectable in oviducts from 9M group (Fig. 1F-G). The findings above delineate a clear transition from early fundamental role to functional maturation across the developmental stages analyzed.

### Identification and characterization of three smooth muscle cell subtypes

**Fig. 2:**
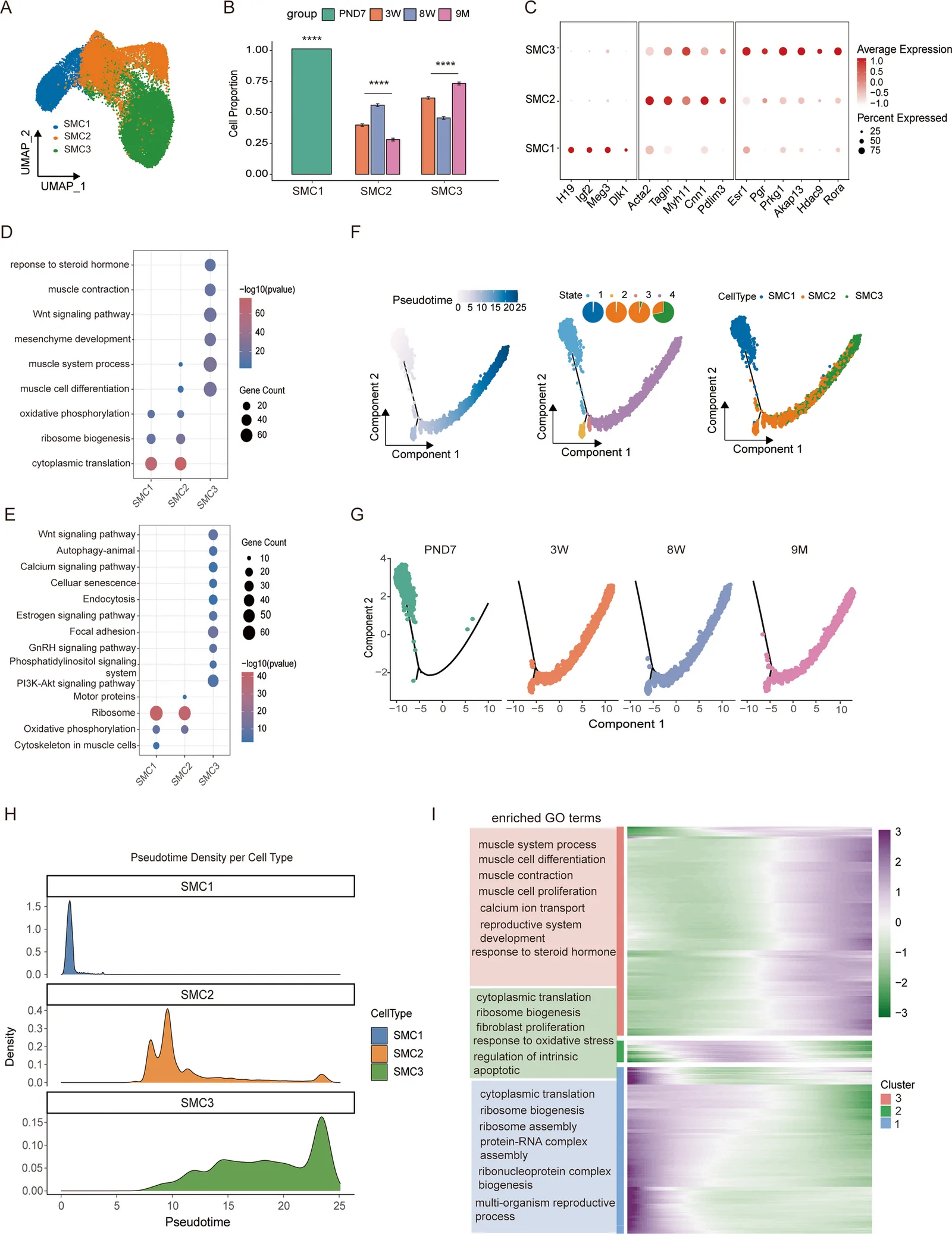
Heterogeneity and developmental trajectory of mouse oviductal smooth muscle cells.** A** UMAP visualization of SMC subclusters. **B** Quantification of SMC subtype proportions at PND7, 3W, 8W, and 9M. Data are mean ± SD (*n* = 3 biological replicates per time point). The Chi-square test with Bonferroni correction was used for pairwise comparisons. SMC1 was only detected at PND7; its proportion was 0% at 3W, 8W and 9M, and the **** above the PND7 bar indicates significant differences between PND7 and each of those stages. For SMC2 and SMC3, all comparisons between 3W, 8W, and 9M (3W vs. 8W, 3W vs. 9M, 8W vs. 9M) reached *P* < 0.0001 and are marked with****. **C** Dot plot heat map showing top markers for SMCs subclusters. **D-E** GO and KEGG pathway enrichment analysis of DEGs in SMCs subtypes (SMC1, SMC2 and SMC3). **F** Pseudotime trajectory of oviduct SMCs plotted by Component 1/2, colored by pseudotime, cell state 1–4 and SMC subtypes. **G** Stage-specific pseudotime trajectories of SMCs at PND7, 3W, 8W and 9M along Component 1/2. **H** Pseudotime density distribution of SMC subtypes. SMC1 exhibits a density peak at early pseudotime, SMC2 peaks at mid-pseudotime, and SMC3 peaks at late pseudotime. **I** Heat map showing the dynamic DEGs along the pseudotime. The related biological process of each cluster is listed on the left

In the oviduct, the smooth muscle layers are referred to as the myosalpinx.Very little information exists on the development of the myosalpinx [13]. SMCs have unclear classifications and changes during developmental timeline. Our UMAP analysis categorized all SMCs into 3 subtypes with 9 cell clusters: SMC1, SMC2 and SMC3 (Fig. 2A and Additional file 4: Fig. S2A; Additional file 5: Tab. S3). Further analysis revealed that SMCs in the oviducts at PND7 were exclusively of the SMC1 subtype, whereas those at 3W, 8W, and 9M comprised both SMC2 and SMC3 (Fig. 2B and Additional file 4: Fig. S2B). SMC1 highly expressed genes such as H19, Igf2, Dlk1 and Meg3 (Fig. 2C). GO and KEGG analysis highlighted enrichment in cytoplasmic translation, ribosome biogenesis and oxidative phosphorylation (Fig. 2D-E), which demonstrated SMC1 may represent a transient, highly active population of embryonic-origin progenitor cells located in the neonatal mouse oviduct. SMC2 exhibited high expression of known genes such as Acta2(αSmooth Muscle Actin, ɑSMA), Tagln, Myh11, Cnn1 and Pdlim3 (Fig. 2C). Pathway analysis revealed that muscle cell differentiation, muscle system process and motor proteins were enriched in subtype 2, consistent with a mature, contractile smooth muscle phenotype (Fig. 2D-E). SMC3 expressed markers including Esr1, Pgr, Prkg1, Akap13 and Rora (Fig. 2C). Functional enrichment analysis revealed that it was significantly enriched for GO terms related to muscle contraction and response to steroid hormone (Fig. 2D). KEGG pathway analysis highlighted the enrichment of autophagy, along with key regulatory pathways, such as PI3K-Akt signaling, Phosphatidylinositol signaling system and endocytosis (Fig. 2D-E and Additional file 6: Tab. S4). To validate these subtypes at the protein level, we performed immunofluorescence staining for IGF2 (SMC1), αSMA (SMC2), and ESR1 (SMC3). IGF2 was detected at PND7, confirming the presence of SMC1 at this stage, whereas αSMA and ESR1 were both present in SMCs from older groups, such as 3W and 8W (Additional file 4: Fig. S2C). These results confirm the existence and spatial distribution of the three SMC subtypes. Furthermore, we noted that multiple autophagy-related DEGs in SMC3 are known interactors or components of a core autophagy-initiating kinase, that is the Class III PI3K (phosphoinositide 3-kinase), Vps34 (Vacuolar Protein Sorting 34) [14]. These included Ambra1(autophagy and Beclin 1 regulator 1, a key activator of autophagy initiation) [15], Pik3r1 (a signaling interactor of Vps34 via mTORC1-mediated crosstalk) [16], Uvrag (a component of the Vps34 complex) [14], and Atg4a (a key molecule synergizing with Vps34) [17](Additional file 6: Tab. S4). This observation implies a potential role of the Vps34-centered regulatory network in modulating SMC3 biological properties during oviduct development.

Next, pseudotime trajectories were performed for SMCs using Monocle2 to investigate dynamic states and cell transitions. The analysis revealed that SMC1 was at the beginning of the trajectory path (Fig. 2F). This early-stage SMC1 was uniquely distributed in PND7 oviducts (Fig. 2G), whereas SMCs in other older samples were primarily at the terminal ends of SMC subtype 2 and 3 (Fig. 2F-G). Dynamic changes in gene expression patterns associated with transitional states were analyze, categorizing SMCs into three clusters. SMC1 was predominantly in cluster 1, with enriched GO terms related to cytoplasmic translation, ribosome biogenesis, multi-organism reproductive process. SMC2 was mainly in cluster 2, characterized by genes involved in cytoplasmic translation, ribosome biogenesis, fibroblast proliferation, regulation of intrinsic apoptotic signaling pathway and response to oxidative stress. SMC3 was mainly in cluster 3, enriched in muscle system process, muscle cell differentiation, muscle contraction, muscle cell proliferation, calcium ion transport, reproductive system development and response to steroid hormone (Fig. 2H-I).

### Transcriptional profiling and functional divergence of SMC subtype 2 and 3 across aging groups

Further analysis revealed that although SMC2 and SMC3 were present across older samples (3W, 8W, and 9M), their relative proportions exhibited dynamic shift with age. The 8W, sexually mature adult group, was predominantly composed of SMC subtype 2. In contrast, both the peripubertal (3W) and aging (9M) groups were dominated by the SMC subtype 3. This terminal state expanded remarkably to 72.8% of all SMCs in the 9M cohort (Additional file 4: Fig. S2D). To investigate aging-related gene expression changes in the two cell types, we conducted a comparative analysis of transcriptomic alterations for each cell subtype between the young and older age groups. The heatmap in Additional file 4: Fig. S2E summarizes the number and overlap of DEGs across comparisons and cell subtypes, with up-regulated DEGs shown in orange and down-regulated in blue. The complete lists of up‑regulated and down‑regulated DEGs are provided in Additional file 7: Tab.S5 and Additional file 8: Tab. S6, respectively. Notably, the DEG analysis highlighted significant differences between oviducts at 9M and those with reproductive function at 3W or 8W stages (Additional file 4: Fig. S2F). From 3 to 8W, downregulated DEGs in SMC2 and SMC3 were associated with focal adhesion, Wnt signaling pathway and MAPK signaling pathway, whereas upregulated DEGs were related to lysosome, cytoskeleton in muscle cells (Additional file 4: Fig. S2F). From 8W to 9M, upregulated DEGs in SMC2 were associated with cytoskeleton in muscle cells, focal adhesion, regulation of actin cytoskeleton, Ras signaling pathway, PI3K-Akt signaling pathway and endocytosis. In parallel, enrichment for these pathways in SMC3 was less pronounced (Additional file 4: Fig. S2F). Notably, despite sharing a similar percentage trend of SMC2 and SMC3, the DEGs analysis highlighted significant differences between 3W oviducts and those from 9M group. KEGG enrichment analysis revealed that the upregulated DEGs in SMC2 and SMC3 were mainly associated with cytoskeletal remodeling, stress responses and altered immune surveillance. The downregulated DEGs were mainly related to ribosome and oxidative phosphorylation (Additional file 4: Fig. S2F). These data suggest that, in the oviduct from 9M mice, SMCs undergo a functional shift, which is distinct from its role in developmental regulation during puberty.

### Vps34 knockout in smooth muscle cells disrupts normal oviduct structure

**Fig. 3:**
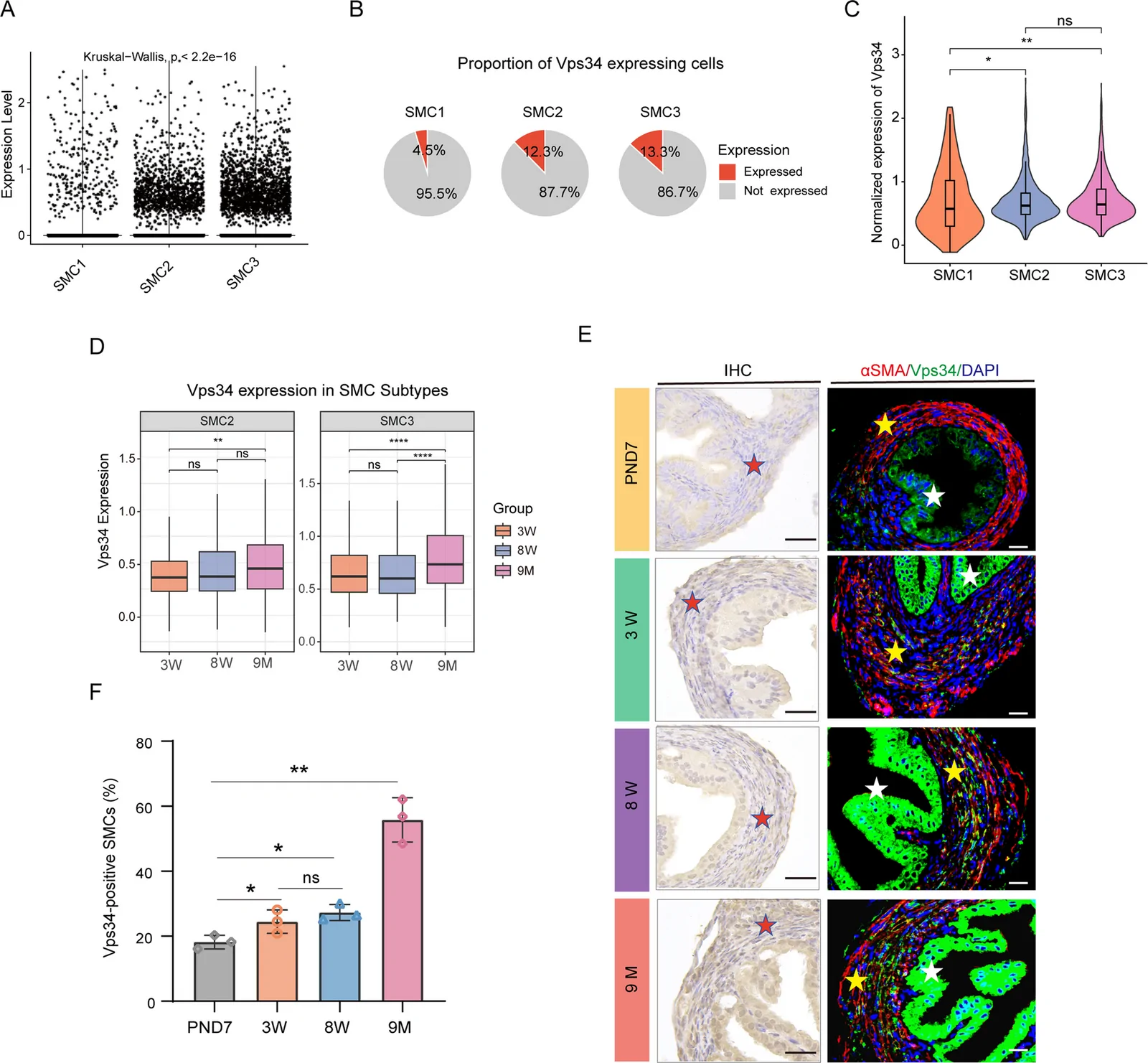
Distinct Expression Patterns of Vps34 in the SMCs. **A** Dot plots showing the expression levels of Vps34 gene across three distinct cell types. Kruskal–Wallis test was performed for overall comparison among the three groups, with a *p*-value < 2.2e-16. Pairwise comparisons between individual subtypes are shown in (**C**). **B** Pie charts depicting the proportion of Vps34-expressing cells within each SMC subtype. **C** Violin plots comparing Vps34 expression between SMC1 vs. SMC2, SMC1 vs. SMC3, and SMC2 vs. SMC3. Statistical significance was determined using the Mann–Whitney U test with Bonferroni correction. *P < 0.05, **P < 0.01, ns: not significant.** D** Vps34 expression levels in SMC2 and SMC3 subtypes at 3W, 8W and 9M were presented by box plots, ** *p* < 0.01, **** *p* < 0.0001, and “ns” means non-significant difference. **E** Representative images showing the spatial distribution and relative expression level of Vps34 among different groups by IHC (left) and immunofluorescence staining (right) of αSMA(red), Vps34 (green). The stars (red and yellow) indicate the location of the smooth muscle layer. The white stars indicate the location of the epithelium. Scale bar = 50 μm. **F** Quantification of Vps34 + SMCs as percentage of αSMA + cells at PND7, 3W, 8W, and 9M. Data are mean ± SD. For each time point, oviduct sections from three mice were analyzed, with five random fields per mouse. Images were processed and quantified using Image J. *p < 0.05; **p < 0.01 (one‑way ANOVA)

Given the observed enrichment of Vps34-interacting autophagy genes in SMC3, we next characterized the expression pattern of Vps34 across oviductal SMC subtypes and further explored its functional role in oviduct development using a conditional knockout mouse model. First, we verified Vps34 expression across all SMC subtypes via scRNA-seq and found that it was expressed in all SMCs, especially in SMC3 (Fig. 3A-B), but with no significant difference in levels between SMC2 and SMC3 (Fig. 3C). Moreover, Vps34 expression exhibited a developmental increase over the maturation timeline, with a higher level detected in oviducts from 9M group (Fig. 3D). To confirm the spatial distribution of Vps34-expressing cells in SMCs, we performed immunohistochemistry (IHC) and observed an age-dependent accumulation of Vps34-positive cells. Immunofluorescence staining also revealed a stronger aggregation of Vps34 signal in the older age groups (Fig. 3E). Quantification showed that the percentage of Vps34 positive SMCs increased from 18.2% ± 2.1% at PND7 to 55.8% ± 6.85% at 9M (Fig. 3F). These results collectively indicate that Vps34 is dynamically expressed in oviductal SMCs which may contribute to oviductal development and function.

**Fig. 4:**
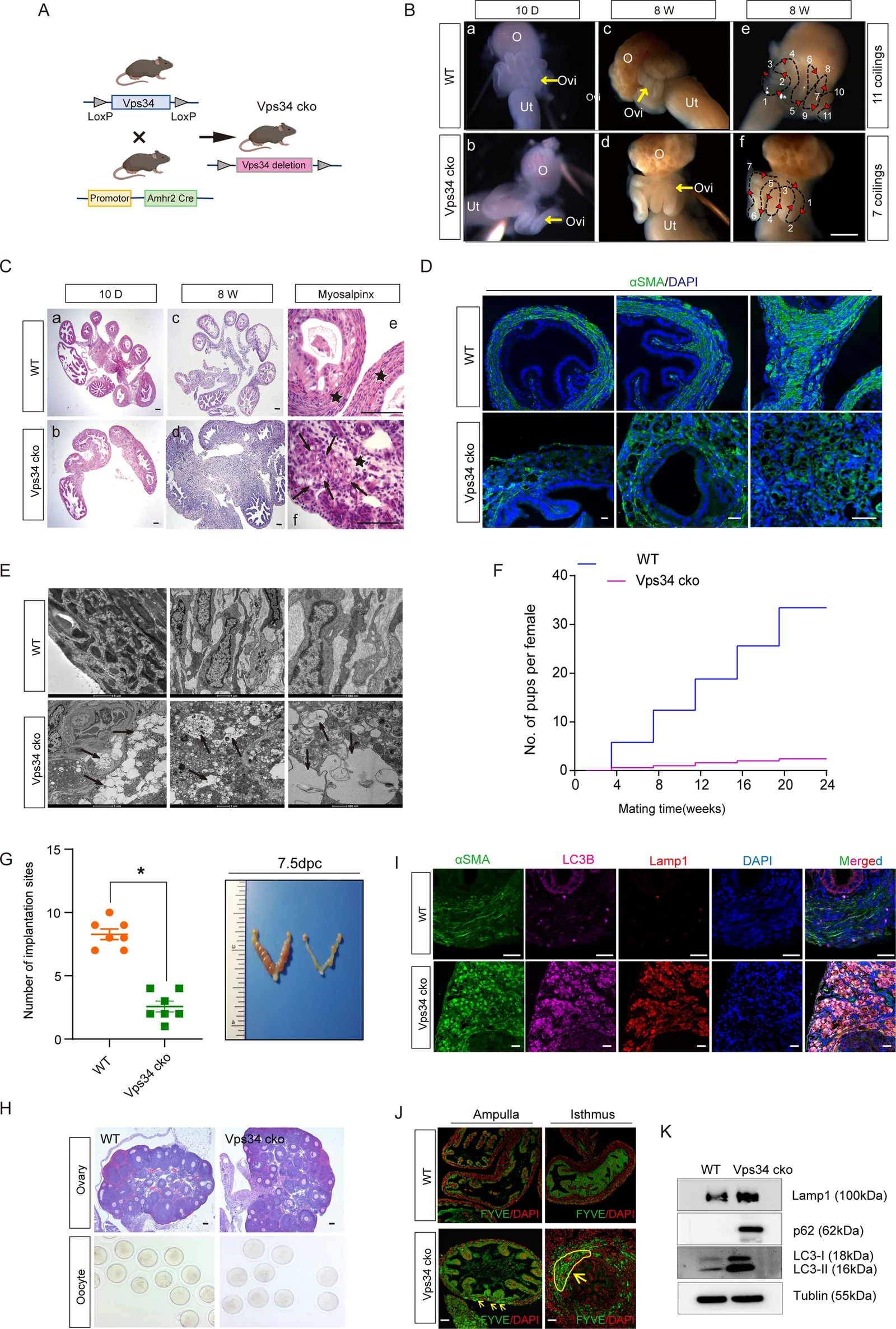
Conditional knockout of Vps34 in SMCs disrupts normal oviduct structure and reproductive capacity.** A** Schematic representation of the deletion of Vps34 in SMCs by using Amhr2-Cre transgenic mice. **B** a-d, Morphological analysis of oviducts in wild-type (WT) and Vps34 knockout (Vps34 cko) mice at 10 days (10 D) and 8 weeks (8 W)(O: ovary; Ut:uterus; Ovi: oviduct); e–h, Representative diagrams of the oviduct coilings. e, Normal mouse oviducts have approximately 11 coils; h, Vps34 cKO oviducts consistently show about 7 coils. This phenotype was fully penetrant (10/10 cKO mice examined). Scale bar = 50 μm. **C** a-d, Hematoxylin and eosin (HE) staining of oviduct tissues corresponding to Ba-d, respectively, Scale bar = 50 μm; e–f, High-magnification HE staining images showing the myosalpinx (smooth muscle layer) of WT and Vps34 cko mouse oviducts. Black stars indicate the smooth muscle layer; arrows highlight vacuoles within the myosalpinx. Scale bar = 50 μm. **D** Immunofluorescence staining of α-smooth muscle actin (αSMA, green) and DAPI (blue) in oviduct tissues of WT and Vps34 cko mice. Scale bar = 50 μm. **E** Transmission electron microscopy (TEM) analysis of oviduct tissues in WT and Vps34 cko mice. Vacuoles was present only in cKO oviducts (100%, *n* = 10 per group) and appeared as diffuse areas. Arrows indicate representative vacuoles. **F** Reproductive curve showing the cumulative number of pups per female mouse in WT(blue) and Vps34 cko (red) groups over a 24-week mating period (*n* = 5 per group). **G** Analysis of embryo implantation sites in WT and Vps34 cko female mice at 7.5 dpc (*n* = 7 per group). **H** Histological and morphological analysis of ovaries and oocytes in WT and Vps34 cko mice. Scale bar = 50 μm. **I-J** Multiplex immunofluorescence staining of oviduct tissues from WT and Vps34 cko mice. Representive images show the localization of αSMA (green, smooth muscle marker), LC3B (magenta, autophagosome marker), Lamp1 (red, lysosome marker), and DAPI (blue, nuclear counterstain). WT SMCs show sparse, scattered LC3B, LAMP1 and FYVE1 puncta; cKO SMCs display large, patch‑like cytoplasmic clusters. Quantification of abnormal clusters (*n* = 5 mice per group, 5 fields per mouse) showed 0/25 fields in WT and 25/25 fields in cKO. Scale bar = 50 μm. **J** FYVE1 domain protein and DAPI staining in the ampulla and isthmus regions of oviducts from WT and Vps34 cko mice. **K** Western blot analysis of autophagy-related proteins in oviduct tissues from WT and Vps34 cko mice

To further investigate the impact of Vps34 on the structure and function of the oviductal myosalpinx, we eliminated the Vps34 gene from mouse SMCs by breeding Vps34^loxP/loxP^ mice with anti-Müllerian hormone type 2 receptor (Amhr2-Cre) transgenic mice (Fig. 4A). As early as 10 days of age, oviducts from Vps34^loxP/loxP^, Amhr2-Cre (+) (Vps34 cKO) mice were significantly shorter than those of WT (Vps34^+/+^) and exhibited reduced number of coilings (Fig. 4Ba-d). As shown in Fig. 4Be-f, normal mouse oviducts have approximately 11 coils [18], in contrast, cKO oviducts consistently showed about 7 coils. Histological examination revealed an abnormal, swollen appearance of the oviductal smooth muscle layer in Vps34 cKO mice compared to WT controls (Fig. 4C). Immunofluorescence analysis of the smooth muscle cell marker, Acta2 (αSMA) in the 8-week-old oviducts showed that SMCs in cKO mice were disorganized compared with age matched controls, characterized by a more irregular and vacuolated arrangement (Fig. 4D). Furthermore, transmission electron microscopy (TEM) confirmed this swollen appearance resulted from intracellular vacuolation, with many vacuoles derived from swollen endosomes and lysosomes (Fig. 4E), similar to phenotypes reported in Vps34 deficient liver and heart [19]. Together, these data indicate that Vps34 deletion causes severe structural damage in the oviductal myosalpinx.

Consistent with the observed structural deficits, these mice also exhibited impaired fertility, as evidenced by a significant reduction in litter size (Fig. 4F-G). To assess whether this fertility defect was due to ovarian dysfunction, we examined ovarian morphology and no obvious differences were found between WT and cKO ovaries (Fig. 4H). Immunohistochemistry showed very low Vps34 expression in ovarian granulosa cells (Additional file 9: Fig. S3A), suggesting that Vps34 deletion in these cells may not be sufficient to impair ovarian function. Accordingly, ovulation and the number of collected MII oocytes were also comparable between WT and cKO mice (Fig. 4H and Additional file 9: Fig. S3B). Additionally, uterine αSMA staining revealed no apparent abnormalities (Additional file 9: Fig. S3C), and the few pups born to cKO mothers were healthy (Additional file 9: Fig. S3D), indicating that the uterus could also maintain basic function in cKO mice. In the oviduct, VPS34 IHC showed reduced signal in the cKO myosalpinx, where the muscle layer appeared disorganized (Additional file 9: Fig. S3E). In contrast, E‑cadherin staining revealed that the epithelial layer remained intact in cKO oviducts (Additional file 9: Fig. S3F).We next examined key autophagy‑related markers. Immunofluorescence staining showed that WT oviduct SMCs contained only sparse, scattered LC3B, LAMP1 and FYVE puncta. In contrast, Vps34 cKO SMCs displayed large, patch‑like cytoplasmic clusters of these proteins, which were never observed in WT samples (Fig. 4I-J). Western blot analysis further confirmed elevated levels of multiple autophagy‑related proteins in cKO oviducts (Fig. 4K). Together, these results suggest that Vps34 deficiency disrupts autophagy homeostasis in the myosalpinx, which may contribute to the structural and reproductive defects observed in cKO mice.

### Identification and functional characterization of ciliated epithelial subtypes

**Fig. 5:**
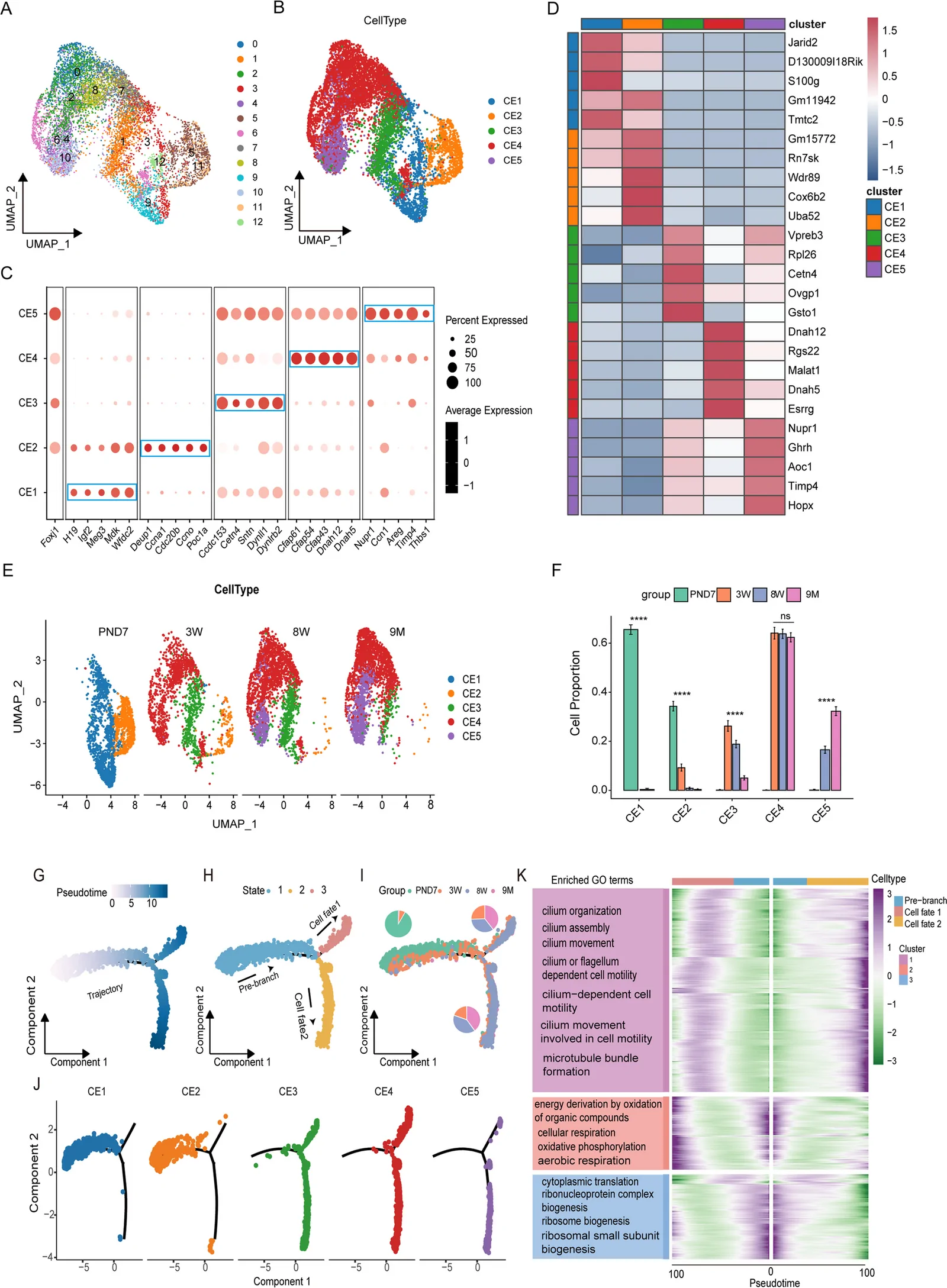
Single-cell transcriptomic profiling of ciliated epithelial cells (CEs). **A** Unsupervised clustering of single-cell transcriptome data identified 13 cell clusters. **B** Results of cell type annotation for the 13 clusters, which were mainly annotated as 5 CEs subtypes. **C** Dot plot showing the representative marker genes across five CEs cell types. **D** Heat map of top 5 DEGs in each CEs subtype. **E** UMAP plots showing the dynamic distributions of five CEs subtypes across different stages. **F** Bar plot quantifying the cell proportion of five CEs subtypes at four developmental stages. Data are mean ± SD from three biological replicates per time point. The Chi‑square test with Bonferroni correction was used for multiple comparisons between all stage pairs (PND7 vs. 3W, 3W vs. 8W, PND7 vs. 8W, 9M vs. 8W, 9M vs. 3W, and 9M vs. PND7). **** indicates that all six pairwise comparisons for a given cell type reached *P* < 0.0001. For CE4, the “ns” means that pairwise comparisons among these three stages (3W vs. 8W, 3W vs. 9M, 8W vs. 9M) were not statistically significant. **G** Pseudotime trajectory analysis of CEs, with the color gradient (light blue to blue) representing pseudotime values (0 to 10) and black arrows indicating the developmental trajectory direction; axes are Component 1 and Component 2 from dimensionality reduction. **H** Trajectory analysis colored by cell state (State 1: blue, State 2: orange, State 3: pink), showing the pre-branch and two cell fate branches (Cell fate 1, Cell fate 2) during CEs development. **I** Trajectory analysis colored by developmental stages (PND7, 3W, 8W and 9M), with pie charts indicating the proportions of cell type at each stage. **J** Pseudotime trajectory analysis of individual CEs cell type, showing the unique developmental trajectory of each subset. **K** Heat map showing the dynamic DEGs along the pseudotime. The related biological process of each cluster is listed on the left

We extended our single-cell analysis to the epithelium populations. Clustering was performed on 26,673 epithelial cells using Ovgp1 and Foxj1 as defining markers of secretory and ciliated epithelial cells [11]. Both ciliated epithelial (CE) and secretory epithelial (SE) contained distinct subtypes. Within the CE cell type, we identify 12 cell clusters and categorized them into 5 major cell subtypes (Fig. 5A-B and Additional file 10: Tab. S7). CE subtype 1, characterized by the expression of progenitor-associated genes, including Mdk, Igf2, Meg3 and H19 (Fig. 5C). CE subtype 2 maintained progenitor features while activating deuterosome-dependent centriole biogenesis (Deup1, Ccno) and cell cycle progression genes (Ccna1, Cdc20b), representing a transitional ciliating population (Fig. 5C). CE subtype 3 strongly expressed genes encoding dynein subunits (Dynll1,Dynlrb2) and structural components Ccdc153 [20, 21], Cetn4 and Sntn [22, 23], yet showed minimal expression of motility-associated Cfap genes (Fig. 5C). In contrast, CE subtype 4 displayed a high-motility signature characterized by outer dynein arm heavy chains (Dnah5, Dnah12) and Cfap complement (Cfap61, Cfap54, Cfap43) (Fig. 5C). We also identified a unique CE subtype 5, which was strikingly defined by the high expression of genes encoding mediators of stress response, cellular senescence and microenvironment remodeling, including Nupr1 [24, 25], Ccn1, Thbs1 [26–28], Areg [29, 30] and Timp4 [31, 32](Fig. 5C). We validated the presence of these CE subtypes by immunofluorescence staining for representative markers (Mdk for CE1, Deup1 for CE2, Dynll1 for CE3, Dnah5 for CE4, and Nupr1 for CE5), as shown in Additional file 11: Fig. S4A. The top differentially expressed genes (DEGs) in each cell type were shown in Fig. 5D. KEGG analysis of the DEGs was showed in Additional file 11: Fig. S4B. Moreover, temporal analysis revealed dynamic shifts during development timeline (Fig. 5E and Additional file 11: Fig. S4C-E). For CE subtype 1, it was only observed at PND7, accounting for approximately 70% of the cells, with subtype 2 cells constituting the remaining 30%. The mature subtypes 3 and 4 comprised ~ 90% of ciliated cells by 3 weeks. Notably, the proportion of CE4 showed comparable percentages at 3W, 8W, and 9M whereas CE3 displayed a gradual decline with age. The stress-responsive CE5 first emerged at the 8W(~ 15% of the CE populations), and increased substantially to 30% by 9M (Fig. 5F and Additional file 11: Fig. S4D-E), revealing continuous epithelial remodeling throughout lifespan.

Next, pseudotemporal analysis reconstructed a developmental trajectory with three major branches: one progenitor-rich branch and two divergent terminal-fate branches (fate 1 and fate 2) (Fig. 5G-H). At PND7, cells were almost exclusively located within the progenitor branch. By 3W, cells had progressed along both terminal branches, and then cells were exclusively distributed along fate1 and fate2 from 8 W through 9M (Fig. 5I). Notably, Fate 1 was highly predominated by subtype 4, in contrast, fate 2 consisted of a mixed population where subtype 4 comprised approximately half of the cells, alongside a substantial proportion of stress-responsive subtype 5 and mature subtype 3 cells (Fig. 5H-J and Additional file 11: Fig. S4F). Heat map showed the DEGs along the pseudotime (Fig. 5K), which revealed three distinct clusters of dynamically expressed genes. GO enrichment indicated their association with core biological functions. Cluster 1 was enriched for terms related to cilium organization, assembly, and motility, including cilium-dependent cell movement and microtubule bundle formation. Cluster 2 was significantly associated with energy metabolism, such as oxidative phosphorylation, cellular respiration, and aerobic energy derivation. Cluster 3 showed enrichment in cytoplasmic translation and ribosome biogenesis, including ribosomal small subunit assembly and ribonucleoprotein complex formation (Fig. 5K).

### Comprehensive characterization of secretory epithelial subtypes

**Fig. 6:**
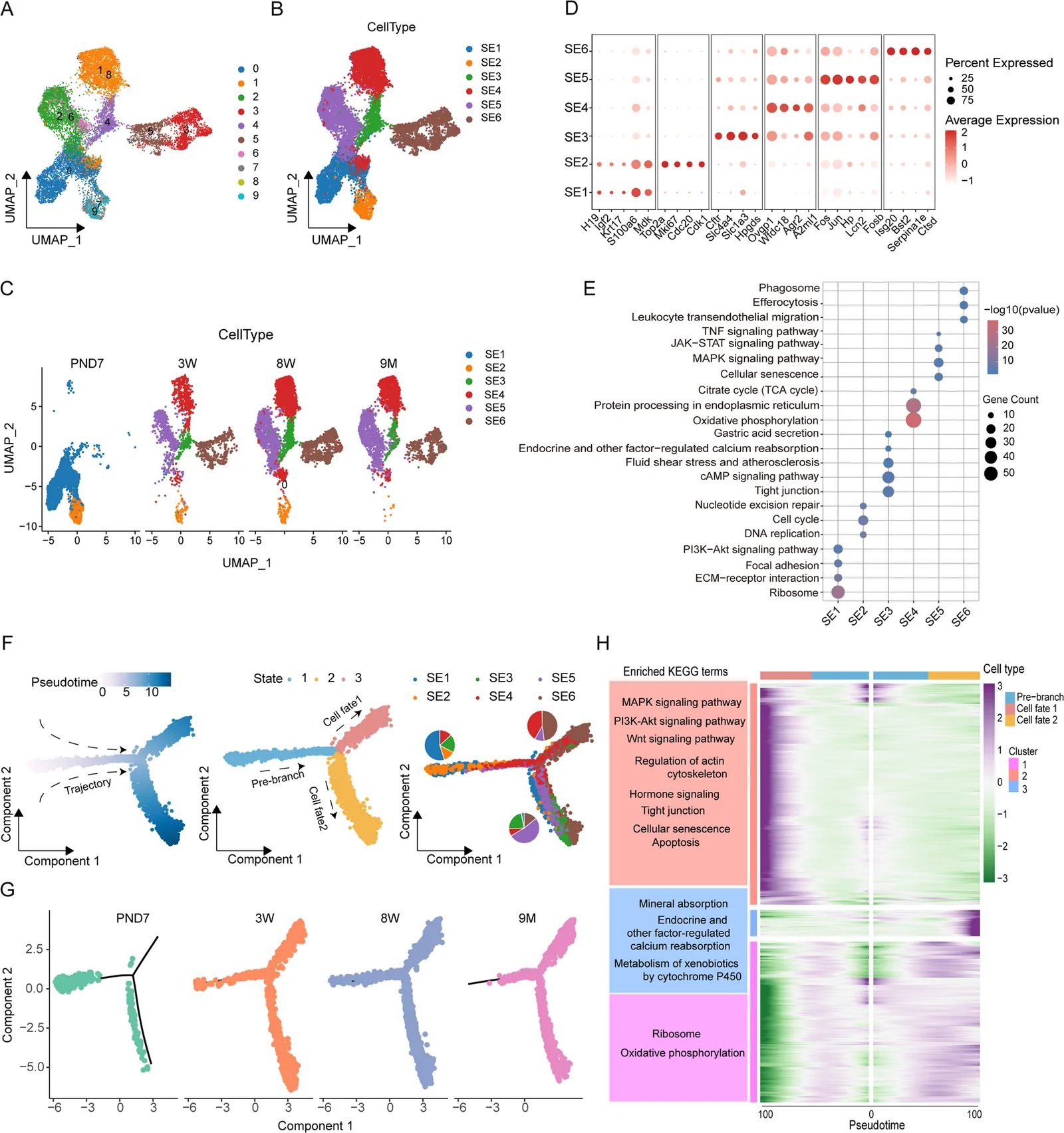
Heterogeneity and developmental trajectory of secretory cell populations(SEs) in the mouse oviduct.** A** UMAP visualization of all epithelial cells, initially clustered into 10 transcriptional subsets. **B** Re-annotation and consolidation of the 10 subsets into 6 distinct secretory cell subtypes. **C** UMAP plots showing the distribution of the 6 SEs across four developmental stages (PND7, 3W, 8W and 9M). **D** Dot plot displaying the expression of canonical marker genes for each secretory subtype. **E** Bubble plot of KEGG pathway enrichment analysis for DEGs among the 6 SEs subtypes. **F** Pseudotime trajectory analysis of SEs, with the color gradient (light blue to blue) representing pseudotime values (0 to 10) and black arrows indicating the developmental trajectory direction; axes are Component 1 and Component 2 from dimensionality reduction (left). Assignment of cells to three states: a pre-branch state and two terminal fate branches (Fate 1 and Fate 2)(Middle). Composition and proportional distribution (pie charts) of the 6 secretory subtypes within the three pseudotime states (Right).** G** Pseudotime distribution of SEs from each developmental stage (PND7, 3W, 8W and 9M). **H** Heatmap depicting the dynamic expression patterns of DEGs along the pseudotime axis, grouped into 3 major clusters. Associated KEGG pathway terms for each gene cluster are indicated on the left

We further categorized all SEs into six subtypes (SE subtypes 1 to 6) with 9 cell clusters (Fig. 6A-B; Additional file 12: Tab. S8). Analysis indicated that SEs in PND7 group were SE subtypes 1 and 2, whereas those in the older age group were mainly SE subtype 3 to 6 (Fig. 6C and Additional file 11: Fig. S4G-H). Further analysis revealed that SE subtype 1 exhibited a progenitor-like signature, with high expression of Krt17, S100a6, Mdk, H19, Igf2 (Fig. 6D). KEGG analysis highlighted enrichment in ribosome, PI3K-Akt signaling, focal adhesion and ECM-receptor interaction pathways (Fig. 6E). SE subtype 2 expressed genes related to cell cycle, including Top2a, Cdk1, Mki67, Cdc20 (Fig. 6D), and enriched in pathways of DNA replication, cell cycle (Fig. 6E). SE subtype 3, marked by Cftr and Slc4a4 (Fig. 6D), constitutes a cellular unit for bicarbonate secretion, which is unequivocally essential for sperm capacitation [33–35]. KEGG pathway analysis corroborated its functional maturity and regulatory complexity. It was significantly enriched in cAMP signaling pathway, the principal activator of CFTR-dependent anion secretion. Additionally, pathways related to gastric acid secretion (a paradigm for epithelial ion transport) and endocrine-regulated calcium reabsorption indicated its core secretion and hormonal control, respectively. Enrichment of tight junction and fluid shear stress pathways further highlighted its potential mechanosensitivity required for directional bicarbonate transport (Fig. 6E). The mature secretory subtype SE4, characterized by expression of Ovgp1, Agr2, Wfdc18, and A2ml (Fig. 6D), exhibited hihgher proporation in oviducts from 8W group (Additional file 11: Fig. S4G-H). This subtype demonstrated enrichment in pathways related to protein processing in endoplasmic reticulum, oxidative phosphorylation, and citrate cycle (TCA cycle) (Fig. 6E). SE subtype 5 displayed a stress-responsive phenotype, and was most abundant in 9M oviducts (Additional file 11: Fig. S4G-H). It was characterized by elevated expression of Jun, Fos, Hp, and Lcn2 (Fig. 6D), with enrichment in pathways associated with MAPK signaling pathway, cellular senescence, apoptosis, and autophagy-animal (Fig. 6E). SE subtype 6 expressed Serpina1e, Isg20, Bst2 and Ctsd (Fig. 6D), and was defined as immunoregulatory secretory cells. Phagosome, leukocyte transendothelial migration and efferocytosis pathways were enriched in SE6 (Fig. 6E). We validated the presence of these SE subtypes by immunofluorescence staining for representative markers (Top2a for SE2, Cftr for SE3, Ovgp1 for SE4, Jun for SE5 and Bst2 for SE6), as shown in Additional file 11: Fig. S4I.

Subsequently, we explored dynamic states and cell transitions in SEs by inferring state trajectories using Monocle 2. The analysis revealed a continuous differentiation path and a Pre-branch node diverging into Cell fate 1 and 2 (Fig. 6F). SE subtype 1 was concentrated at the Pre-branch, serving as the origin of differentiation (Fig. 6F). Cell fate 1 was predominantly populated by SE subtype 6 and 4, while Cell fate 2 was enriched with SE subtype 5 and 3 (Fig. 6F and Additional file 11: Fig. S4J). Based on the trajectory analysis, we next examined the distribution of biological replicates from different developmental stages along the pseudotime continuum (Fig. 6G and Additional file 11: Fig. S4K), illustrating the occupancy of each stage within the inferred differentiation trajectory. Pseudotemporal ordering of the SEs identified three sequential transcriptional states. The early state (Cluster 1), characterized by KEGG pathways related to cellular biosynthesis and energy production, most notably ribosome and oxidative phosphorylation (Fig. 6H). Cluster 2, were enriched for the co-activation of diverse signaling and structural remodeling pathways, included MAPK signaling pathway, PI3K-Akt signaling pathway, Wnt signaling pathway, regulation of actin cytoskeleton and tight junction (Fig. 6H). Cluster 3 switched to an enrichment in pathways governing specialized oviductal functions, such as mineral absorption, endocrine and other factor-regulated calcium reabsorption for ionic homeostasis, and metabolism of xenobiotics by cytochrome P450 (Fig. 6H), highlighting a role in shaping the luminal microenvironment and providing chemical defense.

### Dynamic myoepithelial crosstalk between smooth muscle cells and epithelial subtypes in the developing mouse oviduct

**Fig. 7:**
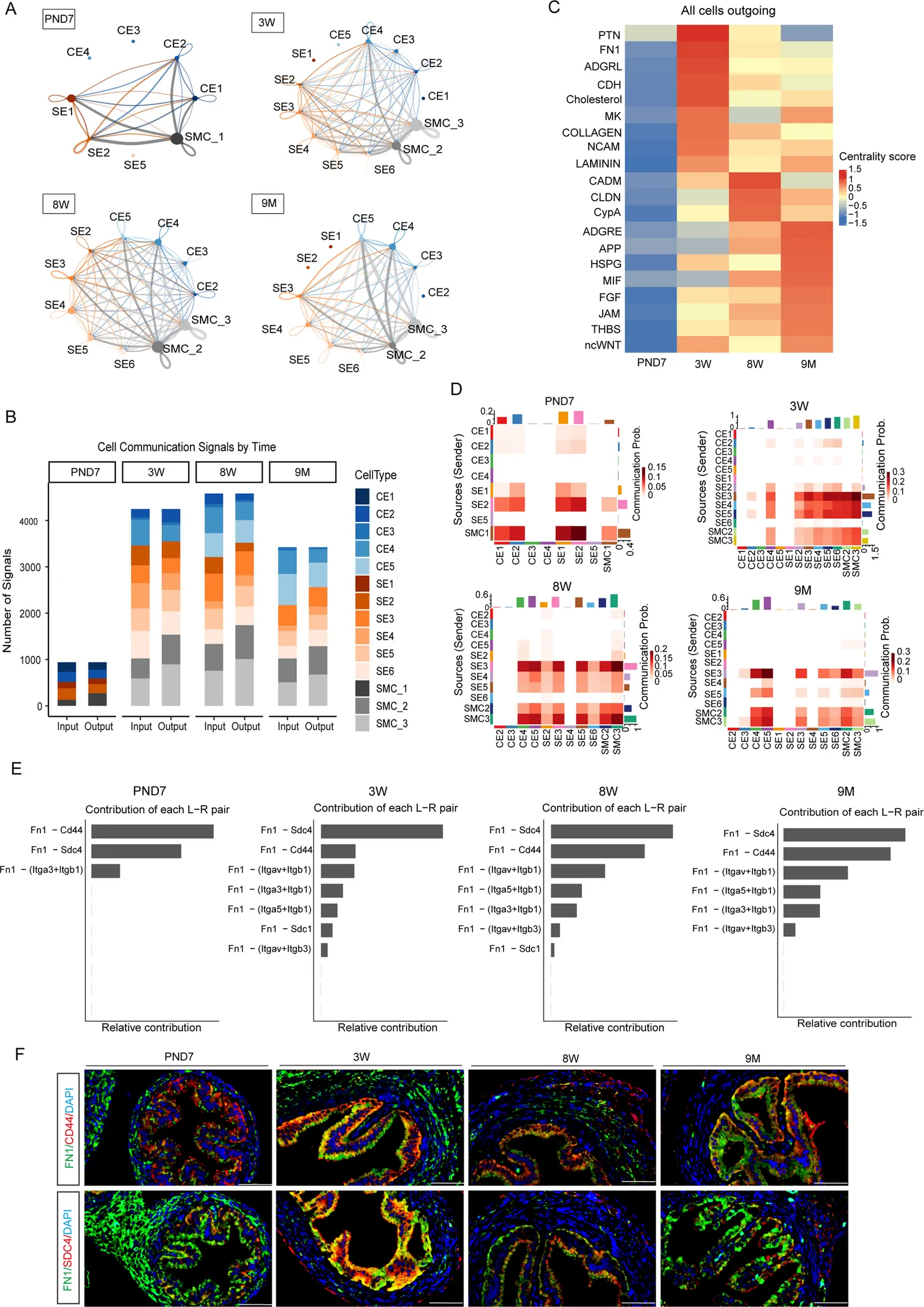
Dynamics of intercellular communication between smooth muscle and epithelial cell subtypes.** A** The strength of intercellular communications between smooth muscle and epithelial cell subpopulations at different stages. Circle sizes, edge width, and edge color represent cell numbers, communication strength, and signaling source, respectively. **B** Stacked bar plot of the aggregated incoming and outgoing signaling number of each smooth muscle and epithelial cell subpopulation during developmental timeline. The bar color is the same as the edge color in (**A**).** C** Heatmap of the outgoing strength of the top 20 signaling pathways derived from the intercellular communications in smooth muscle and epithelial cell subtypes at the four stages. **D** Heatmap depicting the communication probability of the FN1 signaling network across distinct cellular subtypes at four time points. **E** Bar plot illustrating the relative contribution of each ligand-receptor (L-R) pair within the FN1 signaling network specifically between SMCs and epithelial cells at four developmental time points. **F** Immunofluorescence staining images showing the spatial expression patterns of Fn1, Cd44 and Sdc4 in SMCs and epithelial cells in oviducts from the four different stages

We then investigated crosstalk between diverse cell types at each developmental time point by CellChat. Overall, the cell–cell communications within the oviduct was notably enhanced at 3W and 8W, corresponding to periods of key functional maturation (Additional file 13: Fig. S5A-B), especially the incoming and outgoing signals of SMCs (Additional file 13: Fig. S5C). Given the intimate functional coordination between the epithelium and muscular layers in the reproductive tracts [36], we specifically focused on the myoepithelial crosstalk at the two pivotal stages. We found that SMC2 and SMC3 emerged as the most active communicators across all epithelial and smooth muscle cell subtypes (Fig. 7A-B). A deeper dissection of the signaling pathways revealed that it underwent a fundamental shift from 3 to 8W. At 3W, outgoing interactions were dominated by pathways related to extracellular matrix assembly and cellular proliferation, most notably fibronectin (FN1) and pleiotrophin (PTN) (Fig. 7C). By 8W, the signaling landscape transitioned to be dominated by those governing cell adhesion and barrier function, such as CADM and CLDN, alongside sustained immunomodulatory signals (CypA/Ppia) (Fig. 7C). This temporal switch suggests a coordinated developmental program where initial signals drive tissue structuring, which is later supplanted by cues that stabilize tissue architecture and mature functional units. Immunostaining showed the expression of FN1 in the SMCs and epithelial cells of oviducts at four different stages (Additional file 13: Fig. S5Da-d). Co‑staining with α‑SMA confirmed FN1 localization in smooth muscle cells (Additional file 13: Fig. S5De-f).

### Dynamic changes in FN1 ligand-receptor pairs between secretory and smooth muscle cells

To elucidate the mechanism initiating this developmental program, we focused on FN1 signaling by mapping the intercellular communication network and analyzing ligand-receptor (L-R) pairs. Our approach identified FN1 originating mainly from SMCs and SEs, primarily mediated through the Fn1-Cd44 and Fn1-Sdc4 axes (Additional file 13: Fig. S5E-F). We therefore deconvoluted FN1 signaling dynamics across these subtypes to better understand myoepithelial crosstalk. Our results revealed a dynamic, biphasic pattern of FN1 ligand-receptor pairs between secretory and smooth muscle cells: at PND7, SMC1 was the primary FN1 sender (mainly via Fn1-Cd44), while from 3W through 9M, the subtype SE3 became the dominant source, accompanied by a shift in the dominant interaction to Fn1-Sdc4 (Fig. 7D-E). Futhermore, the contribution of SMC3 to FN1 pathway showed an age-dependent increase relative to SMC2 (Fig. 7D). We also demonstrated ligand-receptor pairs of the FN1 signaling sending from SE3 to SMCs and CEs, from SMCs to SEs and CEs, which revealed a systematic shift across development (Additional file 14: Fig. S6A-C), reflecting the change of microenvironment in the oviduct.

Further exploration of cell communication, focusing on the expression of Cd44 and Sdc4 within oviduct. Immunostaining showed the expressions of CD44 and SDC4 were mainly in epithelial cells (Additional file 14: Fig. S6D) and increased protein levels were observed in oviduct from 3W group (Fig. 7F). The results indicated CD44 or SDC4-mediated FN1 signals may play a vital role in cellular communications during development of oviduct.

## Discussion

Despite the established roles of the oviduct in reproduction, the cellular complexity of its muscular layer has remained largely unexplored. In this study, we present a comprehensive analysis of single-cell transcriptomic maps of mouse oviducts across different age groups, extending beyond the epithelium to illuminate the dynamic landscape of the oviductal myosalpinx. Our analysis reveals three functionally distinct SMC subtypes with stage-specific shifts in abundance. SMC1 represents a transient progenitor population restricted to neonatal oviducts, while SMC2 and SMC3 emerge postnatally as contractile and hormone-responsive subtypes, respectively.

Several key findings emerge from our study. First, we delineate subtype-specific transcriptional signatures and show that SMC3 is uniquely enriched in autophagy-related pathways, with age-dependent upregulation of its master regulator, Vps34. This observation suggested a previously unrecognized role for autophagy in SMC homeostasis. We tested this hypothesis using a genetic mouse model with SMC-specific Vps34 deletion, which resulted in myosalpinx disorganization, vacuolar degeneration, and subfertility. Second, beyond the SMC compartment, we identify five ciliated and six secretory epithelial subtypes, revealing dynamic remodeling of the epithelial landscape during aging. Building on recent single-cell atlases of the mouse oviduct epithelium [37–39], our study provides a higher-resolution classification and captures developmental dynamics and age-associated stress-responsive clusters not previously described. Third, systematic mapping of SMC-epithelial crosstalk identifies SMC3 and SE3 as central signaling hubs, with fibronectin (FN1) signaling identified as a candidate pathway that may mediate their interaction.

Our findings challenge the view of the myosalpinx as a homogeneous contractile layer, instead revealing a precisely regulated system composed of functionally specialized SMC subtypes. We propose that SMC2 provides basal tone and rhythmic contraction, while SMC3 integrates hormonal cues to fine-tune contractility for oocyte capture and embryo transit [40, 41]. This functional specialization likely underpins the oviduct's ability to dynamically respond to physiological demands. A particularly striking observation is the identification of Vps34 as a critical regulator of myosalpinx integrity. While Vps34 is well known for its role in autophagy and endolysosomal trafficking [42–44], its function in oviduct morphogenesis has not been previously recognized. Our data show that Vps34 deletion disrupts oviduct coiling, which is a complex and poorly understood developmental process [13, 18, 45]. Prior studies have implicated genes such as Beta-catenin (Ctnnb1), Transforming growth factor beta (TGFβ) and Dicer1 in oviduct differentiation [13]. Here, we establish Vps34 as an additional and indispensable regulator within this morphogenetic program of the oviduct. In addition to its role in SMC homeostasis, Vps34 may also contribute to epithelial-stromal crosstalk. The FN1 signaling axis, which we identify as a candidate mediator of SMC-epithelial communication, undergoes a dynamic switch during development: from FN1-CD44 signaling in early postnatal stages to FN1-SDC4 signaling in the mature oviduct. This FN1-CD44 axis likely facilitates initial matrix organization and tissue patterning [46–48], while the FN1-SDC4 axis is known to confer robust, mechanosensitive adhesion and promote long-term tissue stability on fibrillar matrices [49, 50]. Given that autophagy can regulate FN1 degradation via the lysosomal pathway [51], an intriguing question is whether Vps34-mediated autophagy influences this signaling switch, linking intracellular homeostasis to intercellular communication. We note that our FN1 ligand-receptor analysis is descriptive and does not provide direct functional evidence. Targeted deletion of FN1 or its receptors will be an important direction for future studies to establish causality.

Finally, our epithelial analysis extends previous work on the human fallopian tube [1, 4, 5, 11] by capturing age-associated remodeling, particularly the expansion of stress-responsive clusters (CE5, SE5) in aged oviducts. While our primary focus was on the smooth muscle compartment, these observations provide a foundation for future studies on epithelial aging and its contribution to tubal pathology. Although the Amhr2-Cre line is not strictly SMC‑specific, it remains a standard tool for reproductive mesenchyme, and the phenotypes strongly support an essential role for Vps34 in oviductal smooth muscle. Continued investigation into oviduct biology will be key to uncovering mechanisms of tubal factor infertility.

## Conclusions

Our integrated single-cell analysis provides a developmental roadmap of the mouse oviduct, shifting the focus to its dynamic muscular cells. We define a heterogeneous smooth muscle compartment with age-specific subtypes, establish Vps34-dependent autophagy as a pillar of myosalpinx integrity, and map a finely-timed FN1-mediated dialogue between epithelial and smooth muscle layers. Whether these two pathways are functionally linked, for example, through autophagy-dependent regulation of FN1, remains an important question for future investigation. Overall, our work redefines the oviduct as a complex, cooperatively assembled organ, offering a foundational cellular and molecular framework for future studies on tubal physiology and related reproductive disorders.

## Methods

### Ethics approval and consent to participate

This study involving animals was approved by the Animal Core Facility of Jiangsu Health Vocational College (Nanjing, China) (approval no. JHVC-IACUC-2022-B045; approved 12 November 2022) and was performed following the Guidelines for the Care and Use of Animals of the Experimental Animal Management Committee (Jiangsu, China). The committees adhere to the ethical standards of the International Council for Laboratory Animal Science (ICLAS).

### Animals

Female C57BL/6J mice at four developmental stages: postnatal day 7 (PND7), 3 weeks (3W), 8 weeks (8W), and 9Months (9M) were purchased from Vital River Laboratory Animal Technology Co. (Beijing, China) for single-cell RNA sequencing. Vps34^flox/flox^ mice were purchased from The Jackson Laboratory (019081), and Amhr2-Cre mice were obtained from the Model Animal Research Center of Nanjing University (Nanjing, China). These mice were maintained on identical C57BL/6 J genetic backgrounds. Initially, Amhr2-Cre heterozygotes were bred with Vps34flox/flox mice. Then the resulting male Vps34^flox/+^, Amhr2-Cre mice were bred to female Vps34^flox/flox^mice to generate Vps34^flox/flox^, Amhr2-Cre females. Vps34^flox/flox^, Amhr2-Cre(-) female littermates served as controls. These mice were housed in the Animal Core Facility of Jiangsu Health Vocational College (Nanjing, China). All mice were maintained under a 12 h dark/12 h light cycle at 22℃ with free access to food and water. Animal care and experimental procedures were performed following the guidelines of the experimental animal management committee (Jiangsu, China).

### Tissues dissection and cell suspension

Oviducts were collected from mice at four developmental stages above. Adult female mice (8W and 9M) were monitored by daily vaginal cytology for at least two consecutive estrous cycles. Only mice in the diestrus stage (characterized by a predominance of leukocytes in vaginal smears) were used for tissue collection. PND7 and 3W mice are sexually immature and were collected without cycle staging. For scRNA-seq, three biological replicates were prepared per time point. For PND7, each replicate consisted of oviducts pooled from 6 mice. For 3W, 8W and 9M, each replicate consisted of oviducts from 4 mice. A total of 12 sequencing libraries (3 replicates per time point) were generated. Oviduct tissues were dissected and subjected to enzymatic digestion in 500 µL of HBSS containing 1 mg/mL collagenase I, 1 mg/mL collagenase IV, and 0.01% DNase I at 37 °C for 40 min with gentle agitation. To terminate digestion, 500 µL of HBSS supplemented with 10% fetal bovine serum (FBS) was added. The cell suspension was then centrifuged at 400 × g for 5 min at 4 °C. After complete aspiration of the supernatant, the pellet was resuspended in 500 µL of HBSS. The resulting single-cell suspension was filtered through a 40 μm cell strainer and washed twice with PBS containing 0.04% bovine serum albumin (BSA). Cell viability was determined by 0.4% Trypan Blue exclusion, and viable cell concentration was adjusted to 1000 cells/μl.

### Single-cell RNA library construction and sequencing

DNBelab C Series High-throughput Single-Cell RNA Library Preparation Set (MGI, #940–001818-00 was utilized for scRNA-seq library preparation. In brief, the single-cell suspensions were converted to barcoded scRNA-seq libraries through steps including droplet encapsulation, emulsion breakage, mRNA captured beads collection, reverse transcription, cDNA amplification and purification. cDNA production was sheared to short fragments with 300–500 bp, and indexed sequencing libraries were constructed according to the manufacturer’s protocol. Qualification was performed using Qubit ssDNA Assay Kit (Thermo Fisher Scientific) and Agilent Bioanalyzer 2100. All libraries were further sequenced by the DNBSEQ-T7 sequencing platform at Annoroad Gene Technology (Beijing) Co., Ltd. The sequencing reads contained 30-bp read 1 (including the 10-bp cell barcode 1, 10-bp cell barcode 2 and 10-bp unique molecular identifiers (UMI)), 100-bp read 2 for gene sequences and 10-bp barcodes read for sample index.

### Single-cell RNA sequencing data processing

Single-cell RNA sequencing data were processed using an open-source pipeline provided by MGI (DNBelab C Series scRNA-analysis software, https://github.com/MGI-tech-bioinformatics/DNBelab_C_Series_scRNA-analysis-software). Briefly, raw sequencing data were subjected to sample de-multiplexing, barcode processing, and 3’unique molecular identifier (UMI) counting using default parameters. Processed reads were aligned to the mouse reference genome (GRCm38) using STAR (version 2.5.1b). Valid cells were automatically identified based on the UMI count distribution using the barcodeRanks() function from the DropletUtils package to remove background and low-quality droplets. Gene expression levels were then quantified using PISA to generate a gene-by-cell expression matrix for each library.The resulting gene expression matrices were further analyzed in R (version 4.3.1) using the Seurat package (version 4.4.0). Genes detected in fewer than three cells were excluded. Cells with fewer than 200 or more than 10,000 detected genes, with more than 10% mitochondrial gene expression, or with more than 5% erythrocytic gene expression were removed. After quality control filtering, a total of 102,420 cells from 12 samples were retained for downstream analyses.

### Clustering and annotation

Data integration, cell clustering, and dimension reduction were performed by Seurat (Version 4.4.0) [52] and harmony (Version 1.2.0) [53]. First, we identified 2,000 highly variable genes (HVGs), which were used for the following analysis using FindVariableFeatures() function. Using those HVGs, we scaled data and calculated a principal component analysis (PCA) matrix with the top 20 components by RunPCA() function. Next, we integrated different samples by RunHarmony() function to eliminates technical or batch effect. The cells were then projected into a two-dimension space for visualization by a non-linear dimensional reduction method RunUMAP() in Seurat package. The cells were then clustered by FindClusters() function after building nearest neighbor graph using FindNeighbors() function. The cluster-specific marker genes were identified by FindMarkers() function in Seurat package. Clusters were then classified and annotated based on expressions of canonical markers of particular cell types. Clusters with very low nCount_RNA and nFeature_RNA, and without marker genes, were identified as low-quality cells and removed.The remaining clusters were annotated to cell types based on marker genes expression levels. Once the cell types were determined, each cell type was extracted and the resolution was adjusted for subpopulation subdivision, as described above.

### Differential expression genes (DEGs) identification and functional enrichment

Differential gene expression testing was performed using FindMarkers() function. DEGs were filtered using a minimum absolute value of log(fold change) of 0.25 and a maximum P value of 0.05. GO enrichment analysis was performed using clusterProfiler (Version 4.10.0).

### Trajectory analysis

The single-cell pseudo-time trajectory was generated using the Monocle 2 package in R (Version 2.28.0) [54]. The input was a matrix of gene counts for different cell subpopulations. The newCellDataSet function was applied to create an object with the parameter expressionFamily = negbinomial.size. The differentially expressed genes between subtypes were used for trajectory analysis. The reduceDimension() function with the parameters method = "DDRTree" and max_components = 2 was used for dimensionality reduction. The cells were sorted and visualized using the plot_cell_trajectory() function. Genes that changed with pseudo-time were calculated, sorted in descending order by q-value, and displayed using the plot_genes_branched_heatmap function. Genes were clustered into subgroups based on their gene expression patterns.

### Cell–cell communication analysis

CellChat R package (Version 2.2.0) [55] was used to quantitatively infer and analyze intercellular communication networks. CellChat takes gene expression data as user input to model the probability of cell–cell communication by integrating gene expression with the existing database consisting of known interactions between signaling ligands, receptors and their cofactors. In this paper, cell–cell interactions were analyzed individually for different cell types following the default pipeline. Normalized count data from each condition were used to create a CellChat object and the recommended preprocessing functions were applied for the analysis of individual datasets with default parameters. CellChatDB.mouse was used as the database for inferring cell–cell communication. All categories of ligand-receptor interactions in the database were used in the analysis. Communications involving fewer than 10 cells were excluded.

### Estrous cycle analysis

Vaginal smears were collected on glass slides in 10 lL of 0.9% NaCl at 07:00 ~ 08:00 h each morning. After air-drying, samples were stained with toluidine blue O (Amresco, Solon, OH, USA) for 3 ~ 4 min, and then washed and dried. The four stages of the estrous cycle were determined as previously described by analyzing the proportion of three major cell types (epithelial cells, cornified cells, and leukocytes) [56]. Consistent cycles of proestrus, estrus, metestrus, and diestrus (4 ~ 5 days total) in mice were called ‘regular cycles’.

### Immunohistochemistry (IHC) and immunofluorescence

Samples were fixed in 10% formalin overnight and embedded in paraffin and sectioned to a thickness of 5 µm. For IHC, After de-paraffinization and rehydration, sections were processed for blocking of endogenous peroxidase activity and antigen retrieval pretreatment. IHC analyses were performed using a SPlink Detection Kits (Zhong Shan Jin Qiao) withprimary antibodies at 4 °C overnight. Negative controls were performed by incubation with non-immune IgGs. For immunofluorescence, secondary antibodies were changed to Alexa Fluor 488 goat anti-rabbit and Alexa Fluor 488 goat anti-mouse (Thermo Fisher Scientific), and the nuclei were counterstained with 4,6-diamidino-2-phenylindole (DAPI) for 5 min for observation under confocal microscope. The primary antibodies used are as following, PIK3C3/VPS34(Proteintech #12452–1-AP, China), SDC4 (Proteintech #11820–1-AP, China), ACTA2(αSMA) (Proteintech # 23081–1-AP, China), CD44 (Proteintech # 60224–1-Ig, China), FN1 (Proteintech #15613–1-AP, China), DEUP1 (Proteintech #24579–1-AP, China), DYNLL1 (Proteintech#18130–1-AP,China), MDK (Proteintech # 11009–1-AP, China), CFTR (Proteintech #66928–1-AP, China), TOP2A(Proteintech #24641–1-AP, China), OVGP1 (Proteintech#22324–1-AP,China), DNAH5(Proteintech # 31079–1-AP, China), NUPR1 (Proteintech # 15056–1-AP, China), IGF2 (Proteintech #11601–1-AP, China), JUN (Proteintech # 24909–1-AP, China), BST2 (Proteintech #30118–1-AP, China), LC3B (Cell Signaling Technology, #3868), LAMP1 ((DSHB, 1D4B). The other primary antibodies used for immunofluorescence staining are of the same sources as those used for western blotting.

### Western blot analysis

Proteins were extracted from mouse oviduct tissues. After quantification using a BCA kit, 25 µg protein per sample was used for western blot analysis. In brief, proteins were separated by 10% SDS–PAGE gel and then transferred to polyvinylidene fluoride (PVDF) membranes. After blocking with 5% BSA for 1 h at room temperature, blots were incubated with specific primary antibodies overnight at 4 °C with specific antibodies. Antibodies against P62, LC3B, LAMP1 and β-tubulin were purchased from Cell Signaling Technology. The next day, blots were rewarmed for 1 h and incubated with secondary antibodies for 1 h. Signals were detected using the ECL Plus Western Blotting detection system (Beyotime, China). Tubulin was used as a loading control.

### Transmission Electron Microscopy (TEM)

Mouse oviducts were harvested post-euthanasia, trimmed of adherent tissues, and cut into 1mm^3^ blocks in pre-cooled PBS (pH7.4). Then tissues were fixed in 2.5% glutaraldehyde (0.1 M cacodylate buffer, pH 7.4) at 4 °C for 2 h, rinsed, and post-fixed in 1% OsO₄ for 1 h at 4 °C. After gradient ethanol dehydration and epoxy resin embedding, 60–80 nm ultrathin sections were cut, stained with uranyl acetate and lead citrate, and observed under transmission electron microscopy (Tecnai G2 Spirit BioTwin, FEI, USA).

### Fertility testing

Mating trials were initiated 8 weeks of age for female mice. Male mice at 10 ~ 12 weeks of age with proven fertility were used for mating. During the mating experiment, 5 female mice per group were each housed with one male in a single cage for 24 consecutive weeks. The number of offspring delivered per female was recorded once per week, and is reflected in the reproductive curve.

### Histological analysis

Ovaries used for histological analysis were collected from adult female mice, fixed in 4% paraformaldehyde (PFA) (pH 7.5) overnight at 4 °C, dehydrated, and embedded in paraffin. Paraffin-embedded ovaries were sectioned serially at 5 μm thickness for hematoxylin and eosin (H&E) staining.

### Statistics analysis

Box plots were generated using the R base package and default parameters. Hence, the boxes span the interquartile range (IQR; from the 25th to the 75th percentiles) with the centerline corresponding to the median. The whiskers represent the lowest data point still within 1.5 × IQR of the lower quartile and the highest data point still within 1.5 × IQR of the upper quartile. A wilcoxn test was used to assess differences between two groups, while one-way ANOVA was utilized for comparing differences among multiple groups. For comparison of cell type proportions across different groups, the Chi-square test with Bonferroni correction was applied. Data are presented as mean ± standard deviation (SD) from at least three biological replicates per time point. Pairwise comparisons between groups were performed as specified in the legend of each figure. In all figures, *P* < 0.05 was considered statistically significant and is represented with * for *P* < 0.05, ** for *P* < 0.01, *** for *P* < 0.001, and **** for *P* < 0.0001. “ns” indicates not significant (*P* ≥ 0.05). Data are presented as mean ± SD unless specified otherwise.

## Supplementary Information

Additional file 1: Fig S1. Quality control and initial cell atlas of mouse oviduct scRNA-seq data. (A) Violin plots show the distribution of key quality control (QC) metrics for single-cell RNA sequencing (scRNA-seq) samples across four developmental time points. The evaluated QC metrics include the number of detected genes (nFeature_RNA), total RNA read counts (nCount_RNA), percentage of mitochondrial gene expression (percent.mt), percentage of hemoglobin gene expression (percent.hb), and contamination score. (B) UMAP visualization of singlet cells and doublets (red) among total analyzed cells (nCells = 109923). (C) UMAP plot of the single cell distribution in the oviduct, colored by each sample from different stages. (D) UMAP visualizations of marker gene expression of each cell population. smooth muscle cells: Acta2, Myh11, Tagln, Des; Ciliated epithelial cells: Foxj1, Sntn; Secretory epithelial cells: Ovgp1, Krt18; T cell: Trbc2; Proliferating cells: Mki67; Pericytes cells: Rgs5; Myeloid cells: C1qa; Mesothelial cells: Msln; Blood endothelial cells: Kdr,Vwf; Fibroblast cells: Dcn.

Additional file 2: Tab. S1. Number of cells, UMI and genes in each stage.

Additional file 3: Tab. S2. Markers for each of 11 global cell types of mouse oviduct.

Additional file 4:Fig S2. Identification and developmental dynamics of smooth muscle cell subpopulations. (A) UMAP showing unsupervised clustering of SMCs, with cells partitioned into 10 distinct clusters. (B) UMAP plots displaying the distribution of three SMC subpopulations at four postnatal developmental time points (PND7, 3W, 8W and 9M). (C) Representative immunofluorescence staining of three SMC subtypes in mouse oviducts. IGF2 was detected at PND7. αSMA and ESR1 were present in older groups (3W and 8W). Dashed lines: boundary between epithelium and SMCs. Yellow stars: Epi (epithelium); white stars: SMCs (smooth muscle cells). Scale bar = 100 μm. (D) Stacked bar graph quantifying the proportion of SMC1, SMC2 and SMC 3 at PND7, 3W, 8W and 9M. (E) Heatmaps showing the upregulated (above, orange) and downregulated (below, blue) DEGs for each SMC2 and SMC3 among three groups (8W/3W, 9M/8W, 9M/3W). Gene numbers on the right represents from top to bottom, DEGs shared by the two cell types, DEGs shared by at least two groups, unique DEGs of each cell type in each group. The numbers of unique DEGs are annotated. (F) Representative GO terms of DEGs enrichment among three groups (8W/3W, 9M/8W, 9M/3W) in SMC2 and SMC3. Left, the GO terms of upregulated DEGs. Right, the GO terms of downregulated DEGs. P values were calculated by Fisher’s exact test.

Additional file 5: Tab. S3. Marker List for Smooth Muscle Cell Annotation.

Additional file 6: Tab. S4. Functional KEGG pathway annotation of SMC3 subtype.

Additional file 7: Tab. S5. List of up‑regulated DEGs in SMC2 and SMC3 across 3W, 8W, 9M.

Additional file 8: Tab. S6. List of down‑regulated DEGs in SMC2 and SMC3 across 3W, 8W, 9M.

Additional file 9: Fig S3. Normal ovarian and uterine function in Vps34 cKO mice. (A) Vps34 expression in ovarian sections detected by immunohistochemistry. Red stars indicate oocytes; yellow stars indicate granulosa cells. Scale bar = 50 μm. (B) Quantification of MII oocytes collected after superovulation. No significant difference was observed between WT and Vps34 cKO mice. Data are mean ± SD (*n* = 6 per group). ns, not significant (Student's t-test). (C) Immunofluorescence staining of αSMA in the uterus of WT and Vps34 cKO mice. No obvious structural abnormalities were observed. Scale bar = 50 μm. (D) Representative litters from Vps34 cKO mothers at P17.5 (upper) and PND1 (lower). Pups from cKO mothers appeared healthy. (E) Representative immunohistochemistry images of VPS34 in WT and Vps34 cKO oviducts. In cKO samples, background staining in the damaged areas reflects cellular degeneration associated with Vps34 deficiency. Green stars indicate the epithelium; red stars indicate the smooth muscle layer. (F) Representative immunofluorescence images of E-cadherin in WT and Vps34 cKO oviducts. Green stars indicate the epithelium; yellow stars indicate the smooth muscle layer.

Additional file 10: Tab. S7. Marker List for Ciliated Epithelial Cell Annotation.

Additional file 11: Fig. S4. Characterization and validation of ciliated and secretory epithelial subtypes in the mouse oviduct. (A) Validation of ciliated epithelial (CE) subtypes by immunofluorescence. Representative images were chosen from the oviduct region where each marker gave the most specific staining signal. MDK (CE1) labeled epithelial progenitors at PND7; DEUP1 (CE2) was prominent in the isthmus (PND7). DYNLL1 (CE3) and DNAH5 (CE4) are shown in the ampulla at 8W (representative images); NUPR1 (CE5) is shown in the infundibulum at 9M (representative images). Scale bar = 100 μm. (B) Bubble plot showing GO biological process enrichment analysis of ciliated epithelial cell (CEs) subpopulations (CE1-CE5). (C) Bar plot displaying the absolute cell number of five CEs subpopulations at different postnatal time points. (D) Stacked bar graph illustrating the relative proportion of each CE subtype. (E) Stacked bar graph quantifying the proportion of CE1-CE5 subtypes at PND7, 3W, 8W and 9M. (F) Dimensionality reduction plot showing the distribution of five CEs subtypes. (G) Stacked bar graph quantifying the proportion of six secretory epithelial cell subpopulations (SE1-SE6) at four postnatal time points. (H) Grouped bar plot displaying the relative proportion of SE1-SE6 subpopulations across PND7, 3W, 8W and 9M groups; statistical significance is indicated by *** *p* < 0.001. (I) Validation of secretory epithelial (SE) subtypes by immunofluorescence. Representative images were chosen from the oviduct region where each marker gave the most specific staining signal. TOP2A (SE2) was shown in the ampulla at PND7. CFTR (SE3) and OVGP1 (SE4) were detected in the isthmus and ampulla, respectively, at 8W (representative images). JUN (SE5) and BST2 (SE6) were detected in the ampulla at 9M and 8W, respectively (representative images). Scale bar = 100 μm. (J) Dimensionality reduction plots showing the independent clustering of SE1-SE6 with distinct color coding. (K) Dimensionality reduction plot illustrating the distribution of cells from PND7 (green), 3W (orange), 8W (blue), and 9M (purple) groups, overlaid to show developmental stage-specific clustering patterns.

Additional file 12: Tab. S8. Marker List for Secretory Epithelial Cell Annotation.

Additional file 13: Fig. S5. Landscape of intercellular communications across all cell types in mouse oviduct from PND7 to 9M. (A) The strength of intercellular communications among 11 cell types in the oviducts at different developmental stages. (B) Stacked bar plot of the aggregated incoming and outgoing signaling number of each cell population.The bar color is the same with the edge color in (A). (C) Bar chart showing the number of input (blue) and output (red) cell communication signals of SMCs at four PND7, 3W, 8W and 9M. (D) FN1 expression in the mouse oviduct. a-d: FN1 immunofluorescence in mouse oviducts at PND7, 3W, 8W and 9M. Dashed lines mark the boundary between epithelium and smooth muscle layer. Red stars: epithelium (EPi). White stars: smooth muscle layer (SMC). Scale bar = 50 μm; e–f: Double immunofluorescence staining of FN1 (green) and αSMA (red, smooth muscle marker) in 3W oviducts. e: Low‑magnification; f: High‑magnification view. Scale bar = 100 μm. (E) Heatmap depicting the overview of communication probability across all cell types of the FN1 signaling network. (F) Bar plot illustrating the relative contribution of each ligand-receptor (L-R) pair within the FN1 signaling network at four developmental time points.

Additional file 14: Fig. S6. Identification of ligand-receptor pairs of FN1. (A) The significant ligand-receptor pairs (*P* < 0.01) that contribute to the FN1 signaling pathway sending from SE3 to SMCs and CEs subtypes at 3W, 8W and 9M. (B) The significant ligand-receptor pairs (*P* < 0.05) that contribute to the FN1 signaling pathway sending from SMC2 to SEs and CEs subtypes at 3W, 8W and 9M. (C) The significant ligand-receptor pairs (*P* < 0.05) that contribute to the FN1 signaling pathway sending from SMC3 to SEs and CEs subtypes at 3W, 8W and 9M. The dot color and size represent the calculated communication probability and *p*-value, respectively. *p*-values are derived from one-sided permutation test. (D) Immunostaining showing the expressions of CD44 and SDC4 in the oviducts.

## Data Availability

All data generated or analyzed during this study are included in this article, its supplementary information files, and publicly available repositories. Raw data of scRNA-seq have been deposited in the Genome Sequence Archive (GSA) (https://ngdc.cncb.ac.cn/gsa/search?searchTerm=CRA044564) with the accession codes CRA044564 [57].

## Abbreviations

Vps34: Vacuolar protein sorting 34
FN1: Fibronectin 1
SMC: Smooth muscle cell
FT: Fallopian tube
EP: Ectopic pregnancy
scRNA-seq: Single-cell RNA sequencing
UMAP: Uniform Manifold Approximation and Projection
CEs: Ciliated epithelial cells
SEs: Secretory epithelial cells
DEG: Differentially expressed gene
mTOR: Mammalian target of rapamycin
MAPK: Mitogen-activated protein kinase
PI3K-Akt: Phosphoinositide 3-kinase-Protein kinase B
IHC: Immunohistochemistry
Amhr2-Cre: Anti-Müllerian hormone receptor type 2 promoter-driven Cre recombinase
cKO: Conditional knockout
TEM: Transmission electron microscopy
LC3B: Microtubule associated protein 1 light chain 3 beta
FYVE1: Zinc Finger FYVE-Type Containing 1
LAMP1: Lysosomal associated membrane protein 1
DAPI: 4’,6-Diamidino-2-phenylindole
KEGG: Kyoto Encyclopedia of Genes and Genomes
GO: Gene Ontology
cAMP: Cyclic adenosine monophosphate
Cd44: CD44 molecule
Sdc4: Syndecan 4
